# *C2orf71a/pcare1* is important for photoreceptor outer segment morphogenesis and visual function in zebrafish

**DOI:** 10.1038/s41598-018-27928-7

**Published:** 2018-06-26

**Authors:** Julio C. Corral-Serrano, Muriël Messchaert, Margo Dona, Theo A. Peters, Leonie M. Kamminga, Erwin van Wijk, Rob W. J. Collin

**Affiliations:** 1 Department of Human Genetics, Radboud University Medical Center, Nijmegen, The Netherlands; 2 Radboud Institute for Molecular Life Sciences, Radboud University Medical Center, Nijmegen, The Netherlands; 3 Donders Institute for Brain, Cognition and Behaviour, Radboud University, Nijmegen, The Netherlands; 4 Department of Otorhinolaryngology, Radboud University Medical Center, Nijmegen, The Netherlands; 5 Department of Molecular Biology, Radboud University, Nijmegen, The Netherlands

## Abstract

Mutations in *C2orf71* are causative for autosomal recessive retinitis pigmentosa and occasionally cone-rod dystrophy. We have recently discovered that the protein encoded by this gene is important for modulation of the ciliary membrane through the recruitment of an actin assembly module, and have therefore renamed the gene to *PCARE* (photoreceptor cilium actin regulator). Here, we report on the identification of two copies of the *c2orf71/pcare* gene in zebrafish, *pcare1* and *pcare2*. To study the role of the gene most similar to human *PCARE*, *pcare1*, we have generated a stable *pcare1* mutant zebrafish model (designated *pcare1*^*rmc100/rmc100*^) in which the coding sequence was disrupted using CRISPR/Cas9 technology. Retinas of both embryonic (5 dpf) and adult (6 mpf) *pcare1*^*rmc100/rmc100*^ zebrafish display a clear disorganization of photoreceptor outer segments, resembling the phenotype observed in *Pcare*^−/−^ mice. Optokinetic response and visual motor response measurements indicated visual impairment in *pcare1*^*rmc100/rmc100*^ zebrafish larvae at 5 dpf. In addition, electroretinogram measurements showed decreased b-wave amplitudes in *pcare1*^*rmc100/rmc100*^ zebrafish as compared to age- and strain-matched wild-type larvae, indicating a defect in the transretinal current. Altogether, our data show that lack of pcare1 causes a retinal phenotype in zebrafish and indicate that the function of the *PCARE* gene is conserved across species.

## Introduction

Retinitis pigmentosa (RP, MIM 268000) is an inherited retinal disease (IRD) characterized by progressive visual impairment due to the loss of rod photoreceptors. The prevalence of RP in the population ranges between 1:3,000 and 1:5,000, making it the most common form of IRD. In 2010, we and others reported that mutations in *C2orf71* underlie non-syndromic autosomal recessive RP (arRP)^1,2^. Consecutive studies detected additional disease-causing missense and nonsense mutations along its entire coding sequence in cases with RP or cone-rod dystrophy (CRD)^3–8^. Of interest, *C2orf71* mutations represent a frequent cause of arRP in the Swiss population^9^.

*C2orf71* is almost exclusively expressed in the retina^1^ and codes for a ciliary protein that consists of 1,288 amino acids. In the photoreceptor cell, actin localizes at the base of the outer segments (OS), where OS disk neogenesis is initiated^10^. In *C2orf71*^−/−^ mice, the OS are shortened and disorganized, and are marked by a mislocalization of proteins such as rhodopsin and cone opsins^11^. Recently, we have discovered that the protein encoded by *C2orf71* recruits a ciliary actin dynamics module that modifies the ciliary membrane, and hypothesize that this principle may be the driver of photoreceptor OS disk morphogenesis (manuscript submitted). Hence, we have renamed the *C2orf71* gene to *PCARE* which stands for photoreceptor cilium actin regulator. If *PCARE* plays part of a conserved actin-related function for the process of vision, we may expect a similar function for this gene in a non-mammalian organism.

The zebrafish, *Danio rerio*, is a tropical freshwater fish that is commonly used in IRD research^12–14^. Advantages of using zebrafish for such studies are mainly their short life cycle and the large number of offspring. In addition, their *ex utero* development allows for easy tracking of eye development^15^. Despite being genetically distant to humans, zebrafish eyes share some similarities in morphology and physiology to human eyes. Like humans, zebrafish are also diurnal animals, and their retinas present a similar layered structure^16^. A whole-genome duplication event that occurred during the divergence of the teleost fish^17,18^ made the present day zebrafish genome to contain many duplicated genes, of which over 64 corresponding to genes mutated in IRD, including *pcare*^15^.

Transient morpholino-based knockdown of one of the two copies of *pcare* in zebrafish, *pcare1*, resulted in shortening of photoreceptor outer segments and visual dysfunction^2^. However, over the recent years, the specificity of morpholino-based knockdown studies has been debated, mostly due to non-specific binding that may occur^19^. In addition, the transient nature of the knock-down does not allow to study long-term effects.

Here, we have generated a stable *pcare1* zebrafish mutant using CRISPR/Cas9 technology to investigate the effect of the absence of pcare in a non-mammalian model, and demonstrate that, like in humans, the *pcare* gene is essential for retinal function.

## Results

### *Pcare* is duplicated in the zebrafish genome

**Figure 1:**
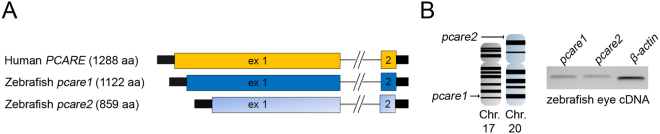
*Pcare* is duplicated in the zebrafish genome. (**A)** Graphical representation of the human *PCARE* gene and the two orthologous zebrafish genes, *pcare1* and *pcare2*. (**B)** Genomic location of the duplicated *pcare* genes in zebrafish. *pcare1* is located in chromosome 17 and *pcare2* is found in chromosome 20 (left). Expression analysis using zebrafish eyes cDNA of *pcare1* and *pcare2* (right). A full image of this gel can be found in the Supplementary Information file (Supplementary Figure S3).

The zebrafish *pcare* paralogues, *pcare1* and *pcare2*, were identified by bioinformatic analysis using the software programs BLAST (https://blast.ncbi.nlm.nih.gov/Blast.cgi) and Ensembl (https://www.ensembl.org/). Reciprocal BLAST searches revealed that both genes are true orthologues of the human *PCARE* gene. Bioinformatic analysis revealed that *pcare1* is located on chromosome 17 and codes for a protein of 1122 amino acids, while *pcare2* is found on chromosome 20 and encodes a protein of 859 amino acids (Fig. 1). A synteny between part of the zebrafish chromosome 17 and the human chromosome 2 on which *PCARE* is located, suggests that *pcare1* is the true orthologue of the human counterpart. To study the expression of these genes, we performed RT-PCR on wild-type zebrafish eye cDNA at 5 days post fertilization (dpf). We observed that both genes are expressed in the zebrafish eye (Fig. 1B). Because of the higher similarity of pcare1 with the human PCARE protein sequence (32.1% of pcare1 vs 25.6% of pcare2, see Supplementary Figure S1) we decided to disrupt the *pcare1* gene.

### Generation of a *pcare1* mutant zebrafish line

**Figure 2:**
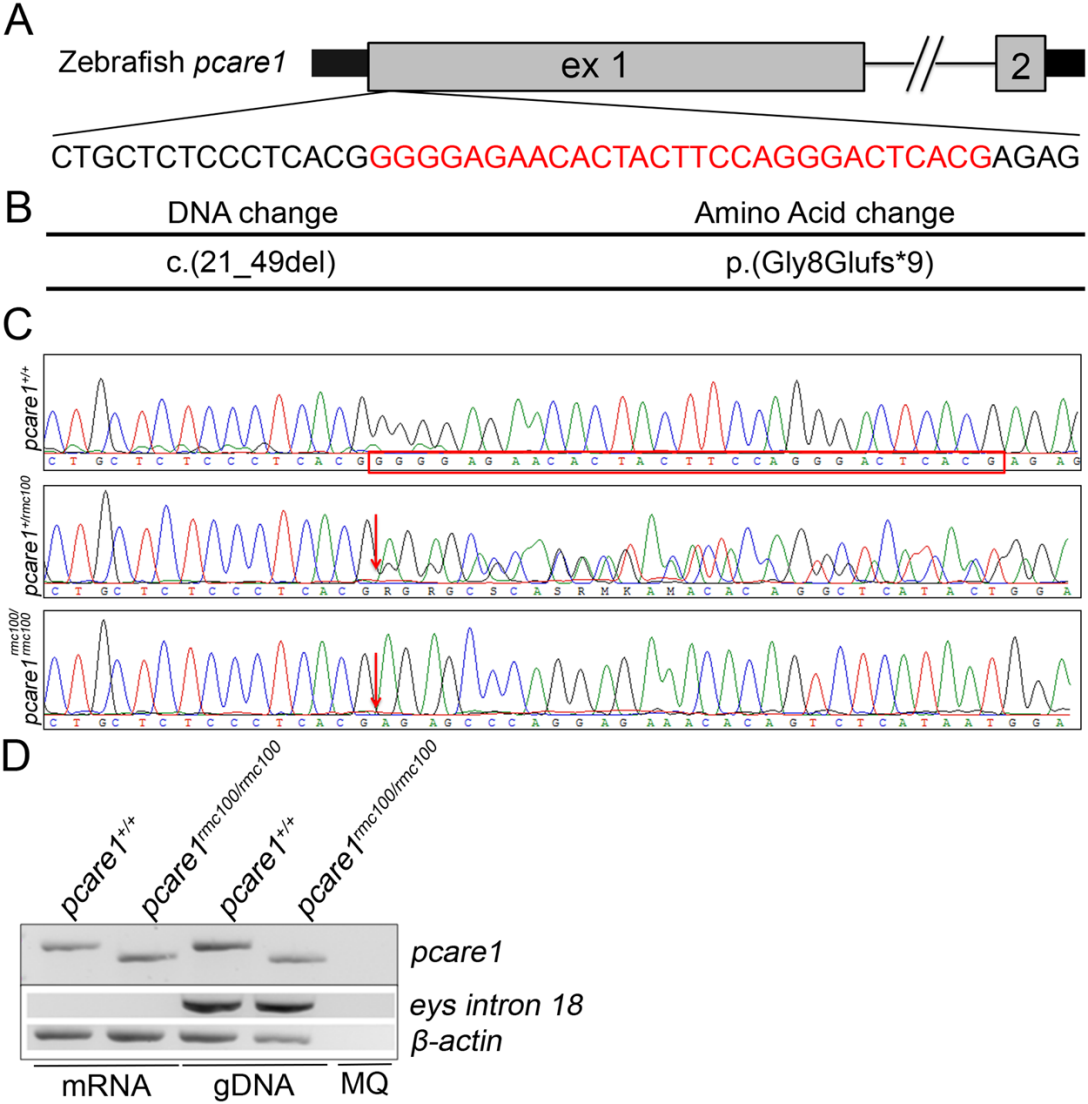
Generation of *pcare1*^*rmc100/rmc100*^ zebrafish. (**A)** The 2 exons of zebrafish *pcare1* gene are shown. In red, the sequence deleted by the CRISPR/Cas9 is indicated. (**B)** The detected DNA change (c.21_49del) leads to a 29 bp deletion causing a frame shift after aminoacid 8 of *pcare1*, predicted to result in a truncated protein (p.Gly8Glufs*9). (**C)** Sequence validation of the targeted region in *pcare1*^+/+^, *pcare1*^+*/rmc100*^ and *pcare1*^*rmc100/rmc100*^ zebrafish. The deleted 29 bp are marked in red in the *pcare1*^+/+^ sequence and absent from the homozygous sequence (red arrows indicate start of the deletion). (D) Upper panel: (RT-)PCR analysis of *Pcare 1* in wild-type and *Pcare1*^*rmc100*/*rmc100*^ zebrafish, showing the presence of a shorter transcript in the mutant fish. Lower panels: analysis of *eys* (only present in genomic DNA) and* β-actin* (present in mRNA and genomic DNA) for comparison and as loading controls.

For the generation of *pcare1* mutant zebrafish, a *pcare1* sequence-specific guide RNA was designed, and co-injected with *Cas9* mRNA into single cell zebrafish embryos. Mosaic fish containing indels around the target region were then out-crossed with wild-type fish to determine germline transmission and the exact genomic lesion that was introduced. We identified four adult F1 fish containing a heterozygous 29-basepair deletion in exon 1 of *pcare1* (c. 21_49del) (Fig. 2A–C). Subsequently, heterozygous *pcare1* carriers were mated to generate homozygous mutant fish, named *pcare1*^*rmc100/rmc100*^, and subsequently crossed for a number of generations. Homozygous mutant fish did not show any signs of gross morphological abnormalities. The 29-basepair deletion is predicted to result in premature termination of translation after amino acid 16 of pcare1 (p.Gly8Glufs*9), most likely resulting in a functional null allele. RT-PCR analysis showed that mutant mRNA is expressed in the *pcare1* mutants, indicating that it does not, or at least not completely, undergo nonsense-mediated decay (Fig. 2D). Staining with different commercial and home-made antibodies targeting the pcare protein in zebrafish did not yield any specific signals, nor any differences between wild-type and *pcare1*^*rmc100/rmc100*^ mutant fish (data not shown). These results suggest that the antibodies targeting human PCARE do not recognize the zebrafish protein, most likely due to the low conservation between the human and zebrafish PCARE sequences (Supplementary Figure S1).

### *Pcare1*^*rmc100/rmc100*^ zebrafish show aberrant photoreceptor morphology

**Figure 3:**
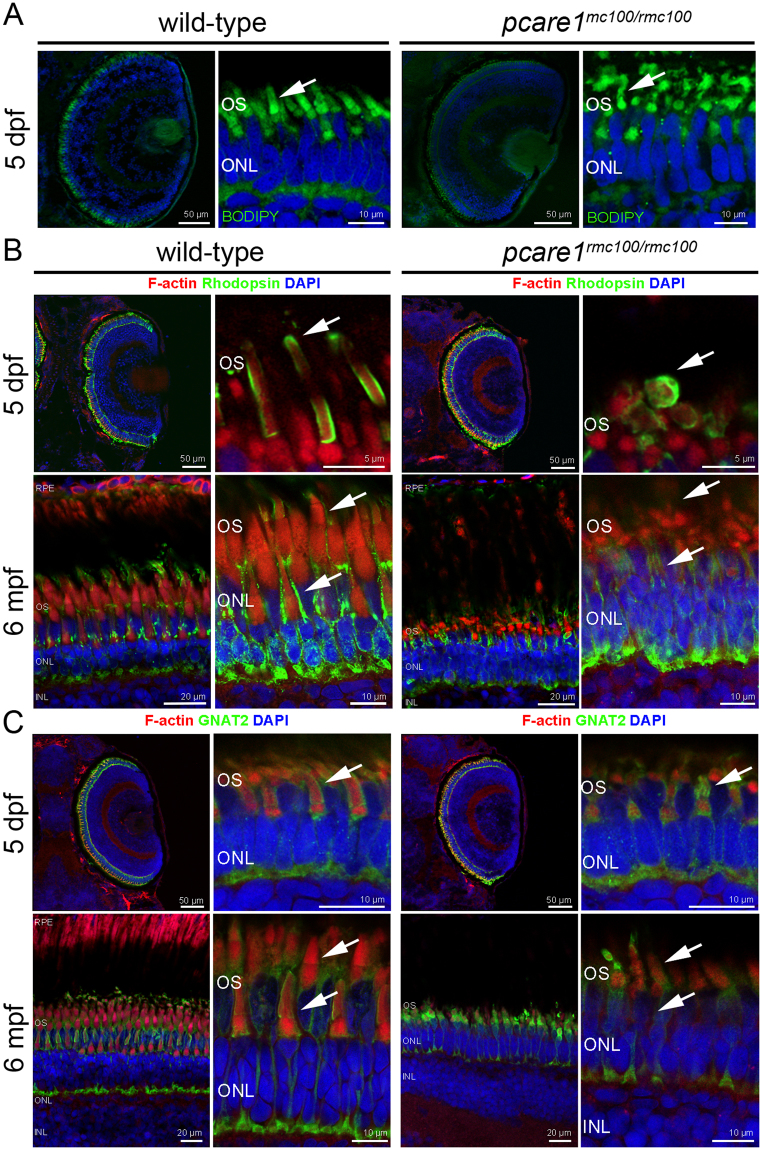
*Pcare1*^*rmc100/rmc100*^ zebrafish show aberrant photoreceptor morphology. (**A)** Analysis of the morphology of the *pcare1*^*rmc100/rmc100*^ zebrafish larval retina (5 dpf) using boron-dipyrromethene (BODIPY) revealed disorganization of photoreceptor outer segments as compared to those of strain- and age-matched wild-type larvae (arrows). (**B)** Siblings of adult zebrafish without (wild-type) or containing (*pcare1*^*rmc100/rmc100*^) the 29 bp deletion in *pcare1* were sectioned and stained with antibodies against F-actin (red), Rhodopsin (green) or, in (**C**) GNAT2 (green). Arrows indicate normal outer segments in control fish and dysmorphic outer segments in mutant fish. Nuclei were stained with DAPI.

To analyze the morphology of the photoreceptor OS, *pcare1*^*rmc100/rmc100*^ and wild-type strain-matched embryos of 5 dpf were sectioned and stained with boron-dipyrromethene (BODIPY). We observed a pronounced dysmorphology of the outer segments in the *pcare1*^*rmc100/rmc100*^ zebrafish as compared to wild-type embryos (Fig. 3A). Since we recently discovered that the human PCARE protein is associated to actin, we also stained retinas, from embryonic (5 dpf) as well as adult (6 months post fertilization, mpf) fish, with an antibody against F-actin. The localization of actin further supported the disorganization of the OS (Fig. 3B,C) in the *pcare1*^*rmc100/rmc100*^ retinas. In addition, both the rod-specific protein rhodopsin and the cone-specific protein Gnat2 exhibited a different pattern in *pcare1*^*rmc100/rmc100*^ fish compared to wild-type fish, presumably affected by the change of morphology of the outer segments (Fig. 3B,C). Additionally, a reduction in the thickness of both outer and inner nuclear layers was observed in *pcare1*^*rmc100/rmc100*^ fish of 6 months of age (Supplementary Figure S2).

### *Pcare1*^*rmc100/rmc100*^ larvae are visually impaired

**Figure 4:**
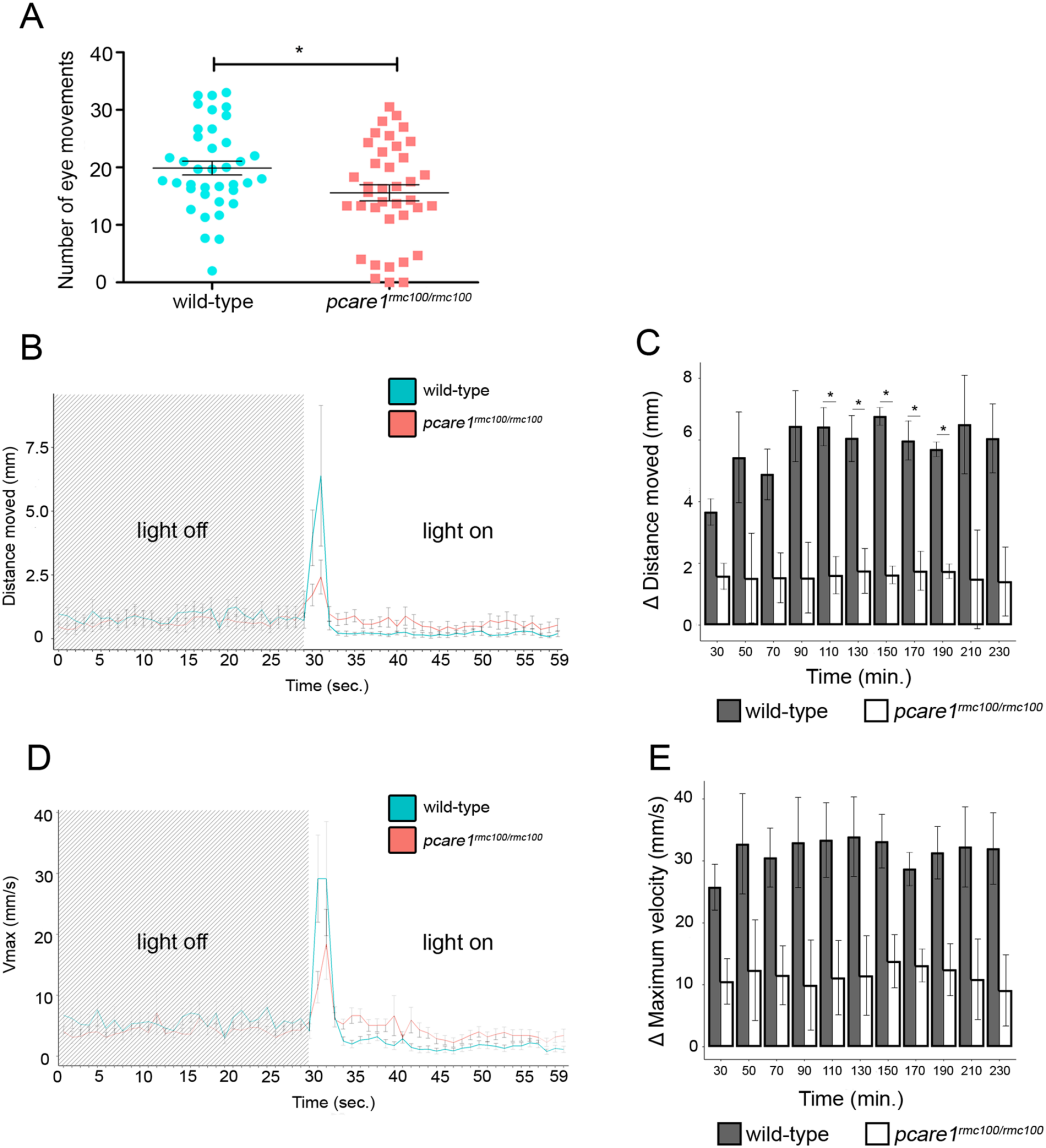
*Pcare1*^*rmc100/rmc100*^ zebrafish are visually impaired. (**A)** Optokinetic response (OKR) measurements revealed a decrease in the number of eye movements of *pcare1*^*rmc100/rmc100*^ larvae (n = 38) compared to control larvae (n = 38). (**B)** Representative graph showing the distance moved (maximum velocity in (**D**)) of the larvae between light-off to light-on change in a one-minute interval. (**C**) Graphs showing the differences in the distance moved (maximum velocity in (**E**)) between wild-type and *pcare1*^*rmc100/rmc100*^ larvae in different time points and combining three independent experiments. Bars indicate the standard error of the mean. P-values are corrected for multiple testing using Benjamini-Hochberg method; the comparisons marked with an asterisk showed statistical significance (*)p-value < 0.05.

**Figure 5:**
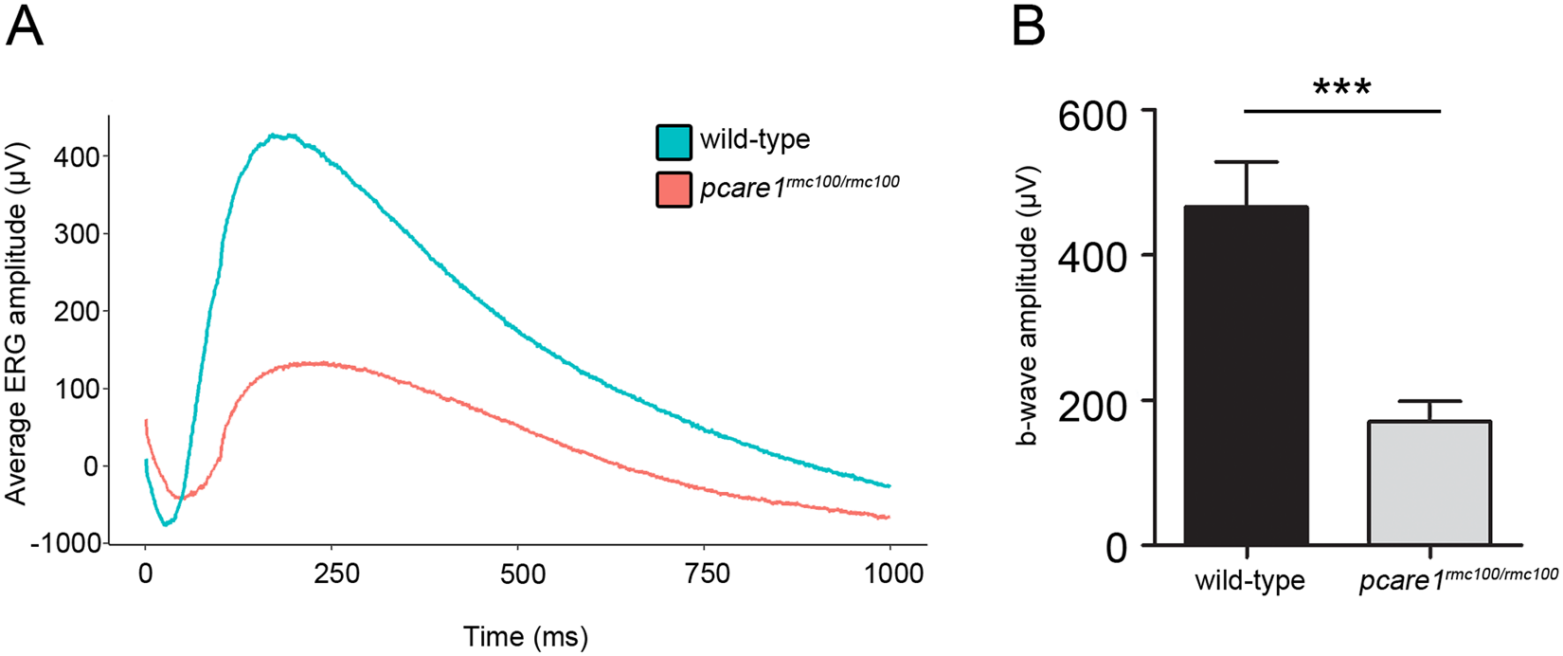
*Pcare1*^*rmc100/rmc100*^ mutant zebrafish show reduced ERG responses. (**A)** The average ERG amplitude to light stimuli of wild-type larvae (n = 15) and *pcare1* mutant larvae (n = 17) was measured. (**B)** Comparison of maximal b-wave amplitudes between wild-type and *pcare1*^*rmc100/rmc100*^ zebrafish larvae. The b-wave amplitude is significantly reduced in *pcare1*^*rmc100/rmc100*^ mutants compared to wild-type larvae; p-value = 0.0004. Bars indicate the standard error of the mean.

To study the response of the zebrafish to visual stimuli, we performed optokinetic response (OKR) and visual motor response (VMR) measurements in wild-type and *pcare1*^*rmc100/rmc100*^ larvae at 5 dpf. OKR analysis, that measures eye movements in a rotating drum, showed a significant decrease in the number of movements in *pcare1*^*rmc100/rmc100*^ larvae compared to wild types (Fig. 4A). Twenty-one percent of the mutant larvae responded with less than 5 eye movements, which can be considered as spontaneous movements, while 5% of the mutant larvae showed no response at all (Fig. 4A). For VMR measurements, we employed the DanioVision system that automatically measures the activity of animals following a series of dark-to-light transitions. Our analysis showed a major decrease of the distance moved after the dark-to-light transitions in *pcare1*^*rmc100/rmc100*^ fish compared to wild-type larvae (Fig. 4B,C). The velocity of the movements was also higher in the wild-type larvae (Fig. 4D,E), though it was not statistically significant. To determine the response of the outer retina to light stimuli, we performed ERG measurements in dark-adapted zebrafish larvae at 5 dpf. The b-wave response, which measures the action potentials of ON bipolar cells^20^, was drastically reduced in *pcare1*^*rmc100/rmc100*^ compared to age-matched control larvae (Fig. 5).

## Discussion

In humans, mutations in the gene *PCARE* (formerly known as *C2orf71*) lead to phenotypes where either the rod photoreceptors, in case of arRP, or the cone photoreceptors, in arCRD, are primarily affected. The outer segments of photoreceptor cells are specialized sensory cilia composed of stacked membrane disks that contain all the necessary elements for phototransduction^21^. Very recently, we have found evidence that PCARE could be an important ciliary actin modulator postulated to drive outer segment disk morphogenesis (manuscript submitted). This study was done using a *Pcare*^−/−^ mouse model and human hTERT RPE-1 cells. Here, we have generated a zebrafish *pcare1* mutant (named *pcare1*^*rmc100/rmc100*^) in order to obtain a better understanding of the function of pcare in a non-mammalian vertebrate. Based on the data presented here, it seems that the function of *PCARE* is conserved, as the photoreceptor outer segments are disorganized in *pcare1*^*rmc100/rmc100*^ zebrafish.

The age of disease onset in patients with *PCARE* mutations ranges from the first to the sixth decade^9^. We observed that *pcare1*^*rmc100/rmc100*^ zebrafish show a worse response than wild-type zebrafish, both in a behavioral assay and after electrophysiological recordings; however, *pcare1*^*rmc100/rmc100*^ zebrafish are not completely blind. These observations correlate with the human phenotype, since patients do not become fully blind until a more advanced age. In mice, disruption of *Pcare* causes severe retinal dystrophy with an early onset, with flat ERG responses already at 8 weeks of age^11^. Behavioral analysis using DanioVision did not show statistically significant difference for velocity and some points of distance moved; however, we can observe a clearly different trend between wild-type and *pcare1*^*rmc100/rmc100*^ larvae. This observation correlates with the high variability in results using visual motor assays that other authors have reported^22^. Additionally, photoreceptive molecules in the zebrafish brain may mediate some of the behavioral responses to light^23,24^.

In our study, *pcare1*^*rmc100/rmc100*^ zebrafish ERG responses show a reduction in the b-wave amplitude as early as 5 dpf, similar to the phenotype observed in mice. Since rod photoreceptors are still not functional in zebrafish at 5 dpf ^25,26^, this indicates that cone photoreceptor cells and/or bipolar cells could be affected by depletion of *pcare1*, although we only measured b-wave responses that are derived from the bipolar cells. In addition, light detection through non-retinal tissues could be accounting for some of the responses.

Because the outer segments are not completely disrupted in *pcare1*^*rmc100/rmc100*^ zebrafish, we hypothesize that other proteins involved in outer segment disk formation may still be functional at their action site. Unlike for other vertebrates, the common ancestor from the teleost lineage underwent a whole-genome duplication event^18^. A consequence of this is the presence of 26,206 protein-coding genes, more than any other sequenced vertebrate species^27^. For some of the duplicated genes, their function has diverged since then^28,29^, while for others it has remained unchanged^30^. Interestingly, parts of zebrafish chromosome 17 and 20, where *pcare1* and *pcare2* are located, descent from the same pre-duplication in the ancestral chromosome. Although disruption of the *pcare1* copy is sufficient to generate a strong retinal phenotype, there is still a possibility that *pcare2* may be involved in a similar process within the photoreceptor cells. Thus, a more severe retinal defect could be expected if both gene copies are disrupted, and future studies will be needed to address the function of pcare2.

Alternatively, other proteins involved in photoreceptor outer segment formation or maintenance could be responsible for our observations. Examples of such proteins are the intraflagellar transport (IFT) protein complex and the motor kinesin-2^31^, which are needed to maintain the extraordinary traffic of proteins along the connecting cilium. Zebrafish knockdown of IFT proteins causes severe retinal degeneration^32^. *Ift57*-deficient zebrafish mutants however are able to form a primitive photoreceptor connecting cilium, rod sacs and flattened outer segment disks, but the disks fail to acquire a proper length and subsequently the photoreceptors die^32,33^. Because of the similarities between the phenotypes of *Ift57* mutants and *pcare1*^*rmc100/rmc100*^ zebrafish mutants, we hypothesize that a similar degenerative process of *pcare1*^*rmc100/rmc100*^ zebrafish outer segments might take place.

Contrary to mammals, where Müller glia cells rarely divide followed by retinal injury, retinal regeneration does occur in zebrafish^34,35^. When injury happens, Müller glia are able to reprogram into retinal stem cells^36^. We however observed a disorganization of the photoreceptor outer segments and degeneration of the outer nuclear layer in *pcare1*^*rmc100/rmc100*^ zebrafish, both at 5 dpf and at 6 mpf. Thus, the regeneration occurring in zebrafish is apparently not sufficient to halt the photoreceptor degeneration caused by absence of pcare1.

In summary, we have generated a *pcare1*-deficient zebrafish line using CRISPR/Cas9 technology. Analysis of the morphology and physiology of embryonic and adult *pcare1*^*rmc100/rmc100*^ zebrafish results in a clear retinal disease phenotype. We provide an additional excellent model organism to study retinal degeneration caused by *PCARE* mutations which will be useful to understand the disease mechanisms. Furthermore, our findings support the hypothesis of the involvement of PCARE in forming and/or maintaining photoreceptor outer segments and, thereby, proper vision.

## Methods

### Ethics statement

Animal experiments were conducted in accordance with the Dutch guidelines for the care and use of laboratory animals, with the approval of the local Animal Experimentation Committee (Dier Experimenten Commissie [DEC]) (Protocol #DEC 2016–0091).

### Zebrafish maintenance

Experimental procedures were conducted in accordance with international and institutional guidelines. Wild-type adult Tupfel Long Fin (TLF) zebrafish were used. Zebrafish eggs were obtained from natural spawning of wild-type breeding fish. Larvae were maintained and raised by standard methods^37^. In brief, embryos were raised in E3 medium (5 mM NaCL, 0.17 mM KCL, 0.33 mM CaCl_2_, 0.33 mM MgSO_4,_ supplemented with 0.1% methylene blue) in a 28 °C incubator with the same day/night cycle (14 h light / 10 h dark). The medium was changed on a daily basis and during the process curved and dead larvae were discarded. Functional and behavioral testing was carried out at 5 days post fertilization (dpf).

### Target site selection and gRNA synthesis

Sites for targeted genome editing were selected using the online software ZiFiT Targeter version 4.2 (http://zifit.partners.org/ZiFiT/)^38,39^. Templates for gRNA transcription were generated by annealing gene-specific oligonucleotides containing the T7 (5′-TAATACGACTCACTATA-3′) promoter sequence, the 20-base target sequence without the PAM (5′-GGGGGAGAACACTACTTCCA-3′), and a complementary region to a constant oligonucleotide encoding the reverse complement of the tracrRNA tail. T4 DNA polymerase (New England Biolabs, Ipswich, Massachusetts, USA) was used to fill the ssDNA overhang and the template was purified using columns (Sigma, St. Louis, Missouri, USA). Transcription of the gRNAs was performed using the MEGAshortscript Kit (Ambion®, Thermo Fisher Scientific, Waltham, Massachusetts, USA).

### Microinjections

Zebrafish embryos were collected and injected at single cell stage with 1 nl of a mix consisting of 4.5 µl gRNA (173,72 ng/ul), 2.5 µl Cas9 protein (2 µg/µl, PNA Bio Inc, Newbury Park, California, USA), 2 µl 1 M KCl and 1 µl 0.5% phenol red dye. To screen for genomic lesions, genomic DNA was extracted from a pool of 15 embryos at 2.5 dpf.

### Genotyping PCR

After injections, genomic DNA was extracted from 2.5 dpf larvae or caudal fin tissue of adult zebrafish. Tissue was incubated in 25 µl (larvae) or 75 µl (fin tissue) lysis buffer (40 mM NaOH, 0.2 mM EDTA) for 20 min at 95 °C. The lysed samples were diluted 10 times after which 1 µl was incubated together with 0.5 µM of the forward and reverse primer, 100 µM dNTPs (Roche, Basel, Switzerland), 0.25 U Taq polymerase (Roche, Basel, Switzerland) and 10 × PCR buffer + 15 mM MgCl2 (Roche, Basel, Switzerland) in a total volume of 25 µl. Samples were denatured at 94 °C for 5 min followed by 35 cycles of amplification consisting of 30 seconds (sec) at 94 °C, 30 sec at 58 °C and 60 sec at 68 °C, followed by a final primer extension of 10 min at 72 °C. To screen for genomic lesions, PCR products were sequenced directly. Primer sequences are listed in Supplementary Table S1.

### Establishment of a *pcare1*^*rmc100/rmc100*^ zebrafish line

Mosaic fish were out-crossed with wild-type TLF fish to determine germline transmission and the exact introduced genomic lesion. Heterozygous mutants were mated to generate homozygous *pcare1* mutants, which were subsequently crossed for a number of generations. A *pcare1*^*rmc100/rmc100*^ zebrafish line was established containing a 29-basepair deletion at the start of exon 1 of *pcare1* (c. 21_49del). This is predicted to lead to premature termination of translation after amino acid 16.

### RT-PCR analysis

To analyze the expression of *pcare1* and *pcare2*, we performed RT-PCR using cDNA extracted from wild-type zebrafish eyes. One microgram of total RNA was incubated with 1 µl iScript Reverse Transcriptase (1708891, Bio-Rad, Hercules, California, USA) and 1x reaction mix in a total volume of 20 µl nuclease free water. For the RT-reaction, the mixture was incubated for 5 min at 25 °C, 30 min at 42 °C and the reaction was stopped by heating at 85 °C for 5 min. PCR analysis was performed as described above. The primers used are listed in Supplementary Table S1.

### Preparation of zebrafish sections

Larvae (5 dpf) were collected in an eppendorf tube and sedated with 2-phenoxyethanol (1:500). After one wash with PBS, embryos or adult eyes (6 mpf) were fixed in 4% PFA overnight, brought gradually to 100% MetOH and stored overnight at −20 °C. After the overnight incubation, samples were rehydrated (75% MetOH in 0.1% PBS-Tween20 (PBS-T), 50% MetOH in 0.1% PBS-Tween20, 25% MetOH in 0.1% PBS-Tween20 for 15 min each), followed by 15 min incubation in 0.1% PBS-Tween20. After this, 10% sucrose in 0.1% PBS-Tween20 was added, followed by an incubation step in 30% sucrose in 0.1% PBS-Tween20 for 1 h at room temperature (RT). Samples were embedded in O.C.T. compound (Tissue-Tek®, 25608-930, VWR, Radnor, Pennsylvania, USA) and snap frozen in isopentane cooled by liquid nitrogen. Cryosectioning was done following standard protocols (seven μm thickness along the lens/optic nerve axis).

### Bodipy staining

To analyze outer segment morphology, cryostat sections of 5 dpf larvae were briefly washed with 1xPBS, permeabilized with 0.5% Triton X-100 in PBS, washed in PBS and incubated for 20 min with TR methyl ester (Bodipy, 1:5000, Life Technologies, Carlsbad, Califoria, USA) and DAPI (1:8000, ITK Diagnostics, Uithoorn, The Netherlands). Sections were washed in PBS, followed by a brief wash with MilliQ and mounted with ProLong(R) Gold antifade reagent (P36930, Life Technologies, Carlsbad, California, USA).

### Immunohistochemistry

Slides were incubated for 2 min in 0.1% PBS-T. Antigen retrieval was done by autoclaving the slides in 10 mM sodium citrate for 1 min at 121 °C. After that, sections were washed three times in 0.1% PBS-T and blocked for 1 h in blocking solution (10% non-fat dry milk in 0.1% PBS-T). Primary antibody (Rhodopsin clone 4D2, mouse, 1:2000, Novus Biological NBP1-48334; GNAT2, rabbit, 1:500, MBL PM075) was incubated in blocking solution overnight at 4 °C. After primary antibody incubation, sections were washed in 0.1% PBS-T, followed by secondary antibody (Alexa Fluor 568® phalloidin, 1:100, Molecular Probes A-12380; Goat IgG, Alexa Fluor 488, donkey, 1:500, Molecular Probes A11055; Mouse IgG, Alexa Fluor 488, donkey, 1:500, Life Technologies A21202) incubation for 45 min in blocking solution. Afterwards, sections were washed in PBS-T and mounted with anti-fade Prolong(R) Gold antifade reagent (P36930, Life technologies, Carlsbad, California, USA). To analyze the thickness of ONL and INL in 6 mpf zebrafish, up to 10 different measurements were taken for each section. Two different sections were analyzed for one wild-type fish and one *pcare1* mutant fish (Supplementary Figure S2).

### Optokinetic response (OKR) assay

All measurements were conducted between 10 am and 5 pm. The OKR was measured as described previously^40–42^. In brief, zebrafish larvae were mounted in an upright position in 3% methylcellulose in a small Petri dish placed on a platform surrounded by a rotating drum. A pattern of alternating black and white vertical stripes was displayed on the drum interior. Larvae (5 dpf) were visualized through a stereomicroscope positioned over the drum. Eye movements were recorded while larvae were optically stimulated by the rotating stripes. Larvae were subjected to a protocol of a 30 sec counterclockwise rotation, a 10 sec rest, and a 30 sec clockwise rotation. Measurements were recorded blinded. Graphs were generated and statistical analysis was done with Graphpad Prism6 software (La Jolla, CA, USA).

### Visual motor response (VMR) assay (DanioVision)

Locomotor activity in response to light-dark conditions was analyzed using the DanioVision system (Noldus B.V., Wageningen, The Netherlands). Larvae were transferred to a 48-wells plate filled with 200 µl E3 medium without methylene blue. Each run consisted of 24 mutant larvae and 24 age-matched control wild-type zebrafish larvae using a 48-wells plate. During the experiment, the temperature was constant at 28 °C using a heating/cooling system (Noldus B.V., Wageningen, The Netherlands). The protocol consisted of 20 min of acclimation (with lid of the system open; room light: 500–650 lux), closing of the lid followed by alternating periods of 10 min dark, 10 min bright light (about 3000 lux) and 10 min dark (in total 12 cycles).

Variables of interest were: *distance moved* (mm) for general activity under dark and light conditions; change in *maximum velocity* (mm∙s-1), to measure the change from dark to light (*maximum velocity* first 30 sec with light minus maximum velocity last 30 sec without light); change in *distance moved* (mm), to measure the change from light to dark (*distance moved* first 30 seconds without light minus *distance moved* from last 30 sec with light)^43–45^. Zebrafish tracks were analysed using the analysis software (EthioVision). First, the missed subjects (<10%) were filtered out. Next, the heat maps of 5-minute interval were plotted, to check for general activity. Fish not moving were removed from the analysis. The distance moved (DM (mm)) per unit of time (1 min, 30 sec or 1 sec intervals) for each animal and group was used as a measure for locomotor activity. In addition, the speed of movement (Vmax (mm/s)) was measured in the dark-to-light transition. The values of 1-second interval were used to study more precisely immediate effects of a sudden change from the dark to the light transition and from the light to dark transition. First, the distance moved was expressed by 30 sec/1 min intervals and compared between the different groups; Second, the more precise per 1 sec interval was used to analyze the direct effects of light/dark and dark/light transitions. EthioVision XT was used to quantify the experiments. Complementary to it, R programming language was used to generate plots, calculate mean values and SEM values, and perform statistical tests. The difference between wild-type and mutant animals was analyzed using two-tailed, unpaired Student’s t-test, and p-values were corrected for multiple testing using Benjamini-Hochberg method. The means vs. standard errors of the mean are shown. Exact p-values can be found in Supplementary Table S2.

### Electroretinograms (ERG)

ERG measurements were performed on isolated larval eyes (5 dpf) as previously described^46^. Larvae were dark-adapted for a minimum of 30 min prior to the measurements and subsequently handled under dim red illumination. The isolated eye was positioned to face the light source. Under visual control via a standard microscope equipped with red illumination (Stemi 2000C, Zeiss, Oberkochen, Germany), the recording electrode with an opening of approximately 20 μm at the tip was placed against the center of the cornea. This electrode was filled with E3 medium (5 mM NaCl, 0.17 mM KCl, 0.33 mM CaCl, and 0.33 mM MgSO4), the same in which the embryos were raised and held. The electrode was moved with a micromanipulator (M330R, World Precision Instruments Inc., Sarasota, USA). A custom-made stimulator was invoked to provide light pulses of 100 ms duration, with a light intensity of 6000 lux. It uses a ZEISS XBO 75 W light source and a fast shutter (Uni-Blitz Model D122, Vincent Associates, Rochester, NY, USA) driven by a delay unit interfaced to the main ERG recording setup. Electronic signals were amplified 1000 times by a pre-amplifier (P55 A.C. Preamplifier, Astro-Med. Inc, Grass Technology) with a band pass between 0.1 and 100 Hz, digitized by DAQ Board NI PCI-6035E (National Instruments) via NI BNC-2090 accessories and displayed via self-developed NI Labview program^47^. All the experiments were performed at room temperature (22 °C). Statistical analysis was performed using GraphPad Prism6 (La Jolla, CA, USA), and graphs were generated in R (Boston, USA) or GraphPad Prism6. For statistical analysis of b-wave amplitude (Fig. 5B), two-tailed unpaired t-test with Welch’s correction was performed; p-value = 0,0004; t = 4.309, df = 19.

## Electronic supplementary material

Supplementary Information

Supplementary Dataset S1

Supplementary Dataset S2
