# Supplementary material for: C2orf71a/pcare1 is important for photoreceptor outer segment morphogenesis and visual function in zebrafish
Source: Sci Rep. 2018 Jun 26;8:9675. doi: 10.1038/s41598-018-27928-7 (PMC6018674; doi:10.1038/s41598-018-27928-7)
Supplement: Supplementary file 1 — Supplementary Information [file 41598_2018_27928_MOESM1_ESM.docx]

***C2orf71a/pcare1* is important for photoreceptor outer segment morphogenesis**

**and visual function in zebrafish**

Julio C. Corral-Serrano^1,2^, Muriël Messchaert^1,3^, Margo Dona^3,4^, Theo A. Peters^3,4^, Leonie M. Kamminga^2,5^, Erwin van Wijk^3,4^, Rob W.J. Collin^1,3,*^

**Supplementary Information**

**Supplementary Figure S1. Sequence alignment of human PCARE protein with zebrafish pcare1, pcare2 and mutant pcare1^rmc100/rmc100^ proteins.**

Identical residues in all sequences are white on a black background, whereas similar amino acids are white on a gray background. Residues that are common for two protein sequences are indicated in black on a light gray background.

PCARE human (1) MGCTPSHS--DLVNSVAKSGIQFLKKPKAIRPGCQGGSERGSIPLLVKNS

pcare1 zebrafish (1) MGCSPSRG--RTLLPGTHESPGETQSHNGSIGDSERGNSTARPYERKFST

pcare2 zebrafish (1) MGCSPSKGQLFSKRPALPSGPAESKKNPNLLSGGDQIQSKAEDSEELAET

pcare1^rmc100^ zebrafish (1) MGCSPSREPRRNTVS-----------------------------------

PCARE human (49) TCYDAGEGLAEEQPSPRRNQTTAKGLCQLMGDPASGKRKDMEGLIPGTKT

pcare1 zebrafish (49) AECEADGAASAKLSTQEININILS--------------------------

pcare2 zebrafish (51) EIEEPAKHSEGKRHSVDDVVCDATAIVR----------------------

pcare1^rmc100^ zebrafish (16) --------------------------------------------------

PCARE human (99) SSSQLNKSQSHMAKDIPFKTQGSHGSQGADFSGDESEESSTQDTSKWKRT

pcare1 zebrafish (73) --------------------------QAKEK-----QEEKREDCEKKGGK

pcare2 zebrafish (79) ------------------EDAAEKILEIISSHDEQRAQETLLEQQEEAVE

pcare1^rmc100^ zebrafish (16) --------------------------------------------------

PCARE human (149) AKCHTSSTQSHCYQTIHPAHEPEGKVDFPEPLVKAHQQAYTYLHSSLSKY

pcare1 zebrafish (92) KSKKSTKSVRVNKRKEKEIKLVQEKVDFPEELVKAHQAAYGYLNPSITKY

pcare2 zebrafish (111) EKKTREKQVKRKKQRKPRLRKNSHVLAKAEFVLIAHQAAYAYLNPSISKY

pcare1^rmc100^ zebrafish (16) --------------------------------------------------

PCARE human (199) EAILCIIHQATQTRELLQPMVSFLLLCFEEISQLLGEISKDGEVLLQEVR

pcare1 zebrafish (142) EDLLGLLDHAAQTQISLQPMVAFMVLRYEEINKGLQEIVEEGEAMFKGNG

pcare2 zebrafish (161) EALLGLLGQAAQTQRSLQTTVASVVLHFEEINQALEDLAADGEQLLREHG

pcare1^rmc100^ zebrafish (16) --------------------------------------------------

PCARE human (249) EDLAWPLKKRE-------------PQEQPNLLQQLLQYTVSKLQVLNGTV

pcare1 zebrafish (192) EHLAWPCEKNKSSYNAKNATTSTCSDPPPDLLQQLLQYTVQRMRQVGQSV

pcare2 zebrafish (211) HNMTWPASLKDYPPTAANGQTG--SPLPSELLQQMLLHSTVNMASMGDSV

pcare1^rmc100^ zebrafish (16) --------------------------------------------------

PCARE human (286) ASLTGSFLEGSSSYLHSTATHLENKLSTKRNVDERLLRALRQLESLASGC

pcare1 zebrafish (242) CGIGDTALEEAVEYFSSITDILDEKLRAKRASESRLMWLLSRIEAASQKK

pcare2 zebrafish (259) RCRSDSALQELAQYFGSMSELIGEKLLAKRAAEERLKQVLCHVEAAAFRK

pcare1^rmc100^ zebrafish (16) --------------------------------------------------

PCARE human (336) GDPGVQGLPLCSEDSGIGADNESVQSVDKLGKQTSWDLAPEPEEWKSVTS

pcare1 zebrafish (292) PSP--EDSALFSEDSGLGAESESLAGSDRQRQRRESSES-----SG--TI

pcare2 zebrafish (309) PGP--EDSALHSEDSGIGAENDCQNGSERQRRSRGSSGS-----------

pcare1^rmc100^ zebrafish (16) --------------------------------------------------

PCARE human (386) PHTEARQSGHTWQQSPFCLGSGRPQDCLLSGAPMAKVQPRAQDEARSPCL

pcare1 zebrafish (333) CATISSPCGFTPIQR----GSYRG--------------------------

pcare2 zebrafish (346) ----GANAGITSAFN-----------------------------------

pcare1^rmc100^ zebrafish (16) --------------------------------------------------

PCARE human (436) SSTSPENITSPPLKLGTSTPCDSFGIGVSVEPHLSKTSRPMDASSLSDSE

pcare1 zebrafish (353) -----R--LLKTMS--SSSSLNSLDSTCTITAKDKKDTDSLLGSVSLDEG

pcare2 zebrafish (357) ------------NS----ASLDQQHASEPVSEDDEDD------EED--DE

pcare1^rmc100^ zebrafish (16) --------------------------------------------------

PCARE human (486) DSSPEEEEEDKMSSMSLCAWQEKTPHSRPQSSPADRESPFQARTRRLRSL

pcare1 zebrafish (394) DFTNGSE---------KVKWNEKRSKQSEASTSELRQ-PRRLPAKRIENP

pcare2 zebrafish (383) DAEPEEE-------------------------------ASGKDELEVQED

pcare1^rmc100^ zebrafish (16) --------------------------------------------------

PCARE human (536) QAQEMILKMKESISERIKFVPVP-------------CGHQDWSEEEEGRT

pcare1 zebrafish (434) QNVEMTLKLKDAISGRIRFLPTQGPGEKAKQTESPKSSSQQWAEDGDKSS

pcare2 zebrafish (402) KKIETTCGFSEAHTSRPAFQGGL--------------QEPAKASYLKRKI

pcare1^rmc100^ zebrafish (16) --------------------------------------------------

PCARE human (573) VVPPRPSTVSGSRRAPERQT----RSQSESCLQSHVEDPTFQELRRVQRD

pcare1 zebrafish (484) KRPQTAASRTSKKKTTVTK-----RSRSADSLRNKAEDPTLIELERTQKE

pcare2 zebrafish (438) RRPKTADNNTLQMKPKHRHLRGPKRSQSAECLCSEEKDSDPHEKLGYQRN

pcare1^rmc100^ zebrafish (16) --------------------------------------------------

PCARE human (619) LSQKLEAFYALGAKGQGQSQEQILQPRAAAVWPNGTCRVSPSNTTSRLKA

pcare1 zebrafish (529) LNQKLERMTKVKGEGN---------KRQCSSKKFLPCQTQNISSVTDRQR

pcare2 zebrafish (488) QHAQHWRRKNCLPEGR----------VRSKIR------------------

pcare1^rmc100^ zebrafish (16) --------------------------------------------------

PCARE human (669) SLTKNFSILPSQDKSILQKCNPHPEDEQGKAGKLPNAIPSGE-VSEAAKA

pcare1 zebrafish (570) SLTRNIFSPSNQRKACNAKVEQATTQNG-------------TEKMDNEKG

pcare2 zebrafish (510) ---GGSSGAPSADRYYG-------LQYG----------------------

pcare1^rmc100^ zebrafish (16) --------------------------------------------------

PCARE human (718) TDWNVRGCPTRTSVKKLIETFSPTESLRMLGDSKDAGASPCLRNC---IM

pcare1 zebrafish (607) KEKDKKAPPVKGSVKIIPVPSPPPSPRQSSGLYRERNSVQKLIDTFSQGL

pcare2 zebrafish (528) ---S--KGPFRAAPPSSPPTFTPEPP--------GRNAVRRLINTFSQGV

pcare1^rmc100^ zebrafish (16) --------------------------------------------------

PCARE human (765) PPRFPKYTGLAPLYPKPQISPASGRESLKMGIGWKPLAPIFPPLPKAEAA

pcare1 zebrafish (657) EESKQVPESVKILGPLKGVRKCGVPIIPGLGP--SGTSAFIDNSILCGQG

pcare2 zebrafish (565) EDSSRQRLLDQRPVRARGHKKCSLPLLQ---------N--SRAALTTGAD

pcare1^rmc100^ zebrafish (16) --------------------------------------------------

PCARE human (815) KSEELSCEMEGNLEHLPPPPMEVLMDKSFASLESPESSKSTENSPKETQE

pcare1 zebrafish (705) ESQCSERTDDLDIDNLPPPPLEVIMDNSFENVQTNAKSENISR-------

pcare2 zebrafish (604) LHLLSDRPEILDLDSLPPPPPEMLMDSSYSSSAGPSAEEGPHDVQCR---

pcare1^rmc100^ zebrafish (16) --------------------------------------------------

PCARE human (865) PGPGEAGPTRRTWASPKLRASVSPLDLLPSKSTASLTKPHSTGPGSGRSS

pcare1 zebrafish (748) ---GRSTLTKKTAMSQKLRASLLSVTVLPSRGNLCKGPVSMSQVCSTQND

pcare2 zebrafish (651) -------------G-QRTLTQR--QPVPLSRANVQRCSISSSRP-----S

pcare1^rmc100^ zebrafish (16) --------------------------------------------------

PCARE human (915) CQPRKPALDLSSPPATSQSPEVKGGTWSQAEKATSLYRQPRKAIAWHHSG

pcare1 zebrafish (795) TREVVKGAHHDSSHETDTESEEAASLYK----------QSRKIIHLRHSS

pcare2 zebrafish (680) RQDAFLGSSIERDYTQVTEG-ENASLYT----------------------

pcare1^rmc100^ zebrafish (16) --------------------------------------------------

PCARE human (965) PPSGQNRTSESSLARP---RQSRERSPPVGRKASPTRTHWVPQADKRRRS

pcare1 zebrafish (835) DSPMEKNTSEQDNRQLSSS----CRSDVGEQKDNSTNETMPNSACRSQNP

pcare2 zebrafish (707) -----------------------------------------KCYP----P

pcare1^rmc100^ zebrafish (16) --------------------------------------------------

PCARE human (1012) LPSSYRPAQPSPSAVQTPPSPPVSPRVLSPPTTKRRTSPPHQPKLPNPPP

pcare1 zebrafish (881) LTSPISRTRVLPSTPLLHRRLPSPPVLKSQPSSSTSSSPPINRKLPSTPS

pcare2 zebrafish (712) TTPPVSRTRLPPSCPSVHHAVPSPPSTTWPPNGRWTPS----AKPHTLPP

pcare1^rmc100^ zebrafish (16) --------------------------------------------------

PCARE human (1062) ESAPAQCKVPSPPTQHPEASPPFSIPSPSPPMSPSQEHKETRDSEDSQAV

pcare1 zebrafish (931) AGQRTLPSTPLMQQEHTPITMTGVTYPFKAPSPPASPKVQRWSRENSTED

pcare2 zebrafish (758) GSQSYLEARAKFCQENQPWPPSCTSTLPRPWGDPARGRVSMGHLQPSGHC

pcare1^rmc100^ zebrafish (16) --------------------------------------------------

PCARE human (1112) IAKVSGNTHSIFCPATSSLFEAKPPLSTAHPLTPPSLPPEAGGPLGNPAE

pcare1 zebrafish (981) TSRVFSNARSVFCPASSSLFEAQSVPTPKPPQAWTS--------------

pcare2 zebrafish (808) PQAHSEPLPDIRAQEGLIEDASDSTSDGTRPECEP---------------

pcare1^rmc100^ zebrafish (16) --------------------------------------------------

PCARE human (1162) CWKNSSGPWLRADSQRRAALCALNPLPFLRRTASDRQPGGRPQPPTLDPT

pcare1 zebrafish (1017) --TGSNVLPRPWGERGRLPVSARGPQPFIRRSQSDRRP--SLSMSSRVPV

pcare2 zebrafish (843) -------------EPADSHLNTLQPQQIAD--------------------

pcare1^rmc100^ zebrafish (16) --------------------------------------------------

PCARE human (1212) STSYESQLGQNSS----SEESPKKDTEPGSSPCSPELQGG-TRRASPPEF

pcare1 zebrafish (1063) ISVAETCGSEPAICTHGLEEGPVREDKIRSEQ---TEIRSAVRSVSHPDL

pcare2 zebrafish (860) --------------------------------------------------

pcare1^rmc100^ zebrafish (16) --------------------------------------------------

PCARE human (1257) CVLGHGLQPEPRTGHIQDKSQPEAQPQQEEVS

pcare1 zebrafish (1110) CIVGQGLQREWEK-------------------

pcare2 zebrafish (860) --------------------------------

pcare1^rmc100^ zebrafish (16) --------------------------------

**Supplementary Figure S2. Measurement of the thickness of outer and inner nuclear layers in *pcare1^rmc100/rmc100^* and wild-type zebrafish at 6mpf.** **A**, Representative images of wild-type and *pcare1^rmc100/rmc100^* zebrafish retinas at 6 mpf. Nuclear layers were stained with DAPI and inverted in grey images. RPE: Retinal Pigment Epithelium; ONL: Outer Nuclear Layer; OPL: Outer Plexiform Layer; INL: Inner Nuclear Layer; IPL: Inner Plexiform Layer; GCL: Ganglion Cell Layer. Scale bars: 20 µm. **B**, Measurements in wild-type and *pcare1^-/-^* zebrafish at 6 mpf shows a significant reduction of both ONL and INL thickness in the *pcare1^-/-^* zebrafish. p-values < 0,01 using a Mann-Whitney U test.


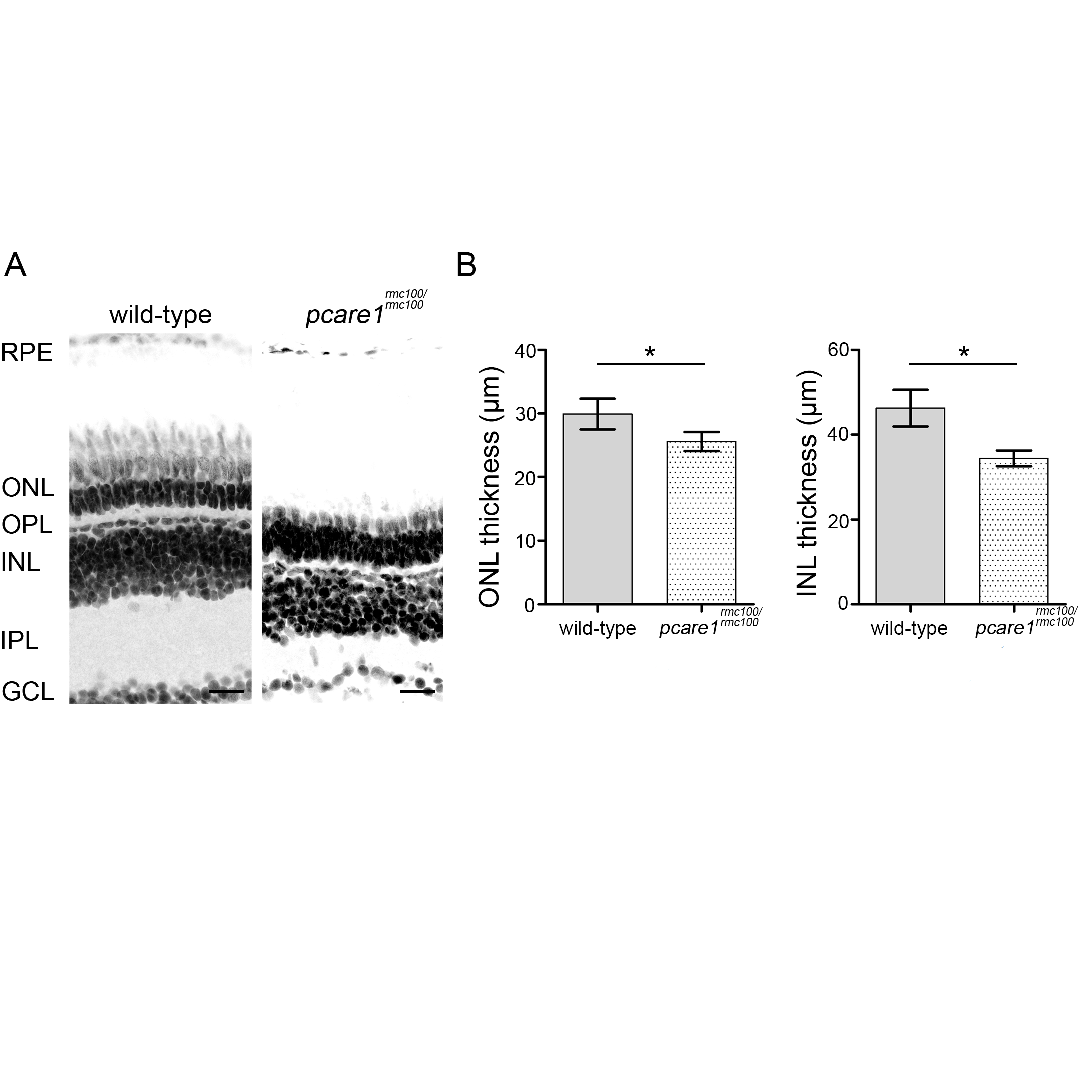


**Supplementary Figure S3.** Full gel picture corresponding to Figure 1B. Marked in red is the cropped region.

**
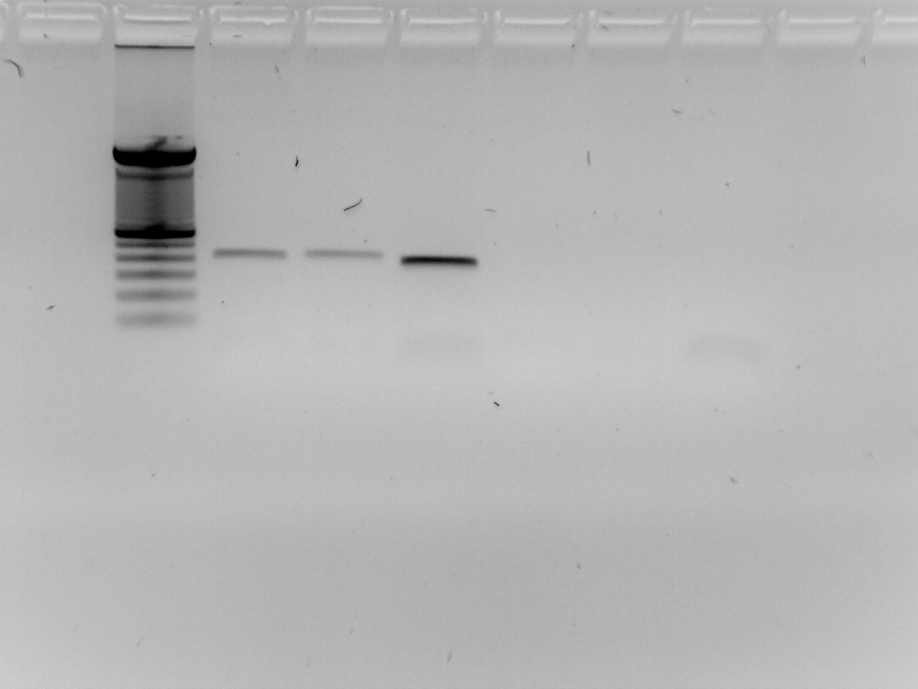
**

**Supplementary Table S1.** Primers used in this study.

| **Primer Name** | **Sequence (5’-3’)** |
| --- | --- |
| pcare1_zebrafish_genotyping_F | TCAATGTGTAACTACTGTGG |
| pcare1_zebrafish_genotyping_R | TTCTTTGACTTCTTTCCTCC |
| pcare1_zebrafish_F | TGGACCTCCACTGGTAGCAATG |
| pcare1_zebrafish_R | CAGCTATTTCATTCCCCAGCAG |
| pcare2_zebrafish_F | TCCAACCACTCCACCAGTCT |
| pcare2_zebrafish_R | GTTCAGGTGAGAGTCTGCCG |

**Supplementary Table S2**. P-values of **A**, ΔDistance moved (mm) and **B**, ΔVmax (mm/s). R programming language was used to generate plots, calculate mean values and SEM values, and perform statistical tests. The difference between wild-type and mutant larvae was analyzed using two-tailed, unpaired Student’s t-test, and p-values were corrected for multiple testing using Benjamini-Hochberg method.

**A,**

| Time point (min.) | Experiment 1 – Average wild types | Experiment 2 – Average wild types | Experiment 3 – Average wild types | Experiment 1 – Average *pcare1^rmc100/rmc100^* mutants | Experiment 2 – Average *pcare1^rmc100/rmc100^* mutants | Experiment 3 – Average *pcare1^rmc100/rmc100^* mutants | p-value | Adjusted p-value |
| --- | --- | --- | --- | --- | --- | --- | --- | --- |
| 30 | 4.489031 | 3.417126 | 3.099486 | 2.977334 | 0.766855 | 1.027767 | 0.076637 | 0.084301 |
| 50 | 7.982758 | 5.421336 | 2.889139 | 2.966498 | 0.637896 | 0.951405 | 0.09942 | 0.09942 |
| 70 | 6.509943 | 4.097312 | 4.064718 | 2.596576 | 0.773006 | 1.246267 | 0.032556 | 0.05864 |
| 90 | 8.29358 | 6.715224 | 4.338462 | 2.303252 | 0.884807 | 1.39785 | 0.038115 | 0.05864 |
| 110 | 7.61268 | 5.575122 | 6.094735 | 2.01309 | 0.445177 | 2.39469 | 0.0049 | 0.017968 |
| 130 | 7.515253 | 5.567596 | 5.10233 | 2.183769 | 1.133339 | 1.964503 | 0.016343 | 0.035954 |
| 150 | 7.343226 | 6.447082 | 6.517712 | 1.394266 | 0.940354 | 2.536749 | 0.001805 | 0.017968 |
| 170 | 4.840743 | 7.028744 | 6.056858 | 2.460793 | 0.942638 | 1.85287 | 0.007478 | 0.020563 |
| 190 | 5.800346 | 5.243898 | 6.054689 | 2.121457 | 0.840032 | 2.275547 | 0.004418 | 0.017968 |
| 210 | 9.109013 | 6.809481 | 3.597526 | 1.60152 | 0.221808 | 2.653249 | 0.071066 | 0.084301 |
| 230 | 8.23621 | 4.533497 | 5.385639 | 1.619625 | 0.701135 | 1.9175 | 0.042647 | 0.05864 |

**B,**

| Time point (min.) | Experiment 1 – Average wild types | Experiment 2 – Average wild types | Experiment 3 – Average wild types | Experiment 1 – Average *pcare1^rmc100/rmc100^* mutants | Experiment 2 – Average *pcare1^rmc100/rmc100^* mutants | Experiment 3 – Average *pcare1^rmc100/rmc100^* mutants | p-value | Adjusted p-value |
| --- | --- | --- | --- | --- | --- | --- | --- | --- |
| 30 | 32.90559 | 21.00921 | 23.42852 | 18.3454 | 6.149363 | 7.165309 | 0.04644 | 0.073936 |
| 50 | 48.34021 | 28.71532 | 21.14943 | 20.63709 | 7.536889 | 8.933083 | 0.111795 | 0.111795 |
| 70 | 39.96277 | 24.55223 | 27.03901 | 18.93714 | 5.513233 | 10.22137 | 0.03933 | 0.073936 |
| 90 | 46.33764 | 31.22294 | 21.29981 | 13.46355 | 8.134596 | 8.308291 | 0.080041 | 0.088046 |
| 110 | 45.24621 | 29.19242 | 25.68748 | 14.22291 | 4.724764 | 14.50949 | 0.046076 | 0.073936 |
| 130 | 46.59508 | 29.04535 | 26.0247 | 12.64319 | 9.390405 | 12.43636 | 0.069475 | 0.084914 |
| 150 | 41.43107 | 31.15287 | 26.87081 | 15.17567 | 10.33201 | 15.93244 | 0.03246 | 0.073936 |
| 170 | 26.16697 | 33.99695 | 26.01176 | 17.57142 | 6.420388 | 15.40455 | 0.024947 | 0.073936 |
| 190 | 39.15427 | 24.80746 | 30.02824 | 14.69152 | 3.367739 | 19.32694 | 0.041441 | 0.073936 |
| 210 | 43.72703 | 31.82563 | 21.26107 | 9.284293 | 3.57503 | 19.82126 | 0.06193 | 0.084914 |
| 230 | 43.39386 | 24.82262 | 27.74842 | 9.85108 | 5.58792 | 11.89881 | 0.04705 | 0.073936 |
